# Case report: Jejunal diverticulosis with chronic interstitial and mesenteric adhesions, chronic mesenteric volvulus, and decompensated small-bowel obstruction

**DOI:** 10.1016/j.ijscr.2024.109549

**Published:** 2024-03-19

**Authors:** Sarlote Agate Vanka, Janis Rudzitis, Ingus Skadins, Juta Kroica, Sintija Miluna-Meldere, Janis Dreijers

**Affiliations:** a Faculty of Medicine, Riga Stradins University, Dzirciema street 16, Riga LV-1007, Latvia; b Department of Surgery, Riga Stradins University, Dzirciema street 16, Riga LV-1007, Latvia; c Department of Biology and Microbiology, Riga Stradins University, Dzirciema street 16, Riga LV-1007, Latvia

**Keywords:** Jejunum, Diverticulosis, Obstruction, mesenteric volvulus, Diagnostic laparoscopy, Case report

## Abstract

**Introduction:**

Jejunal diverticulosis has not gained significant attention because of its rarity and typically asymptomatic course as well as the relative diagnostic inaccessibility of the jejunum. This report presents a rare case of jejunal diverticulosis complicated with chronic interstitial and mesenteric adhesions, chronic mesenteric volvulus, and decompensated small-bowel obstruction.

**Presentation of case:**

An 84-year-old man was admitted to the emergency room with a 24-h history of acute colicky abdominal pain. He denied other signs or symptoms. The preoperative diagnosis based on physical and radiologic evaluations was challenging, and *only diagnostic laparoscopy revealed extensive small-bowel diverticulosis. Midline laparotomy was performed as definitive surgery, revealing* diverticulosis in the proximal 2-m section of the jejunum, starting approximately 20 cm from Treitz's ligament; the affected section was resected. The postoperative recovery was excellent. The histopathologic report confirmed substantial jejunal diverticulosis with chronic fibrosis, adhesions, and strictures.

**Discussion:**

Histopathologic evaluation is necessary because tumors can be misdiagnosed as diverticula. This case report should serve as a reminder for surgeons to be cognizant of the signs of uncommon conditions, such as jejunal diverticulosis.

**Conclusion:**

Albeit rare, jejunal diverticulosis should be considered in the differential diagnosis of acute abdomen.

## Highlights

• Jejunal diverticulosis has not gained significant attention.
• Diverticular disease is markedly less common in the small bowel than in the colon.
• Laparoscopy revealed multiple diverticula in the proximal small intestine.
• The affected small bowel was resected to prevent complications.
• Histopathologic evaluation is necessary because tumors can be misdiagnosed as diverticula.

## 1 Introduction

Despite its long history, jejunal diverticulosis has not gained significant attention among clinicians because of its rarity and typically asymptomatic course as well as the relative diagnostic inaccessibility of the jejunum [1]. Here, we present a rare case of jejunal diverticulosis with chronic interstitial and mesenteric adhesions, chronic mesenteric volvulus, and decompensated small-bowel obstruction to illustrate that jejunal diverticulosis should be considered in the differential diagnosis of acute abdomen. This case has been reported along with SCARE criteria [2].

## 2 Case report

**Fig. 1:**
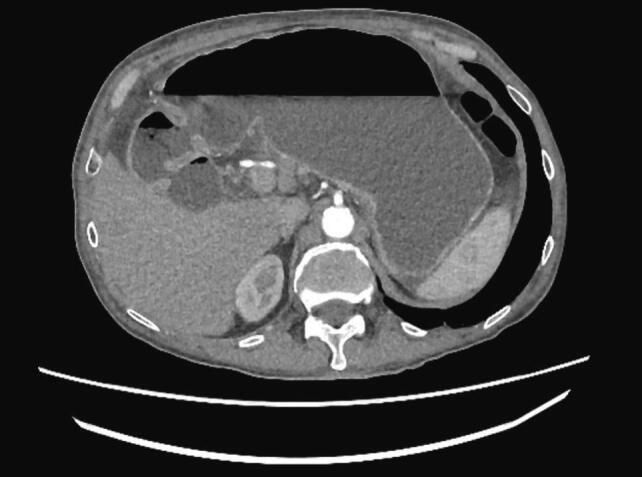
Dilated stomach with a large air-fluid level showed on abdominal computed tomography (CT).

**Fig. 2:**
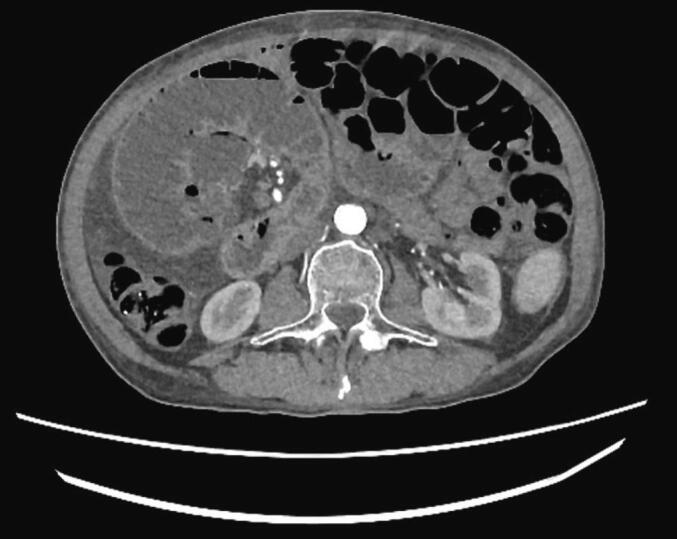
The first loop of the jejunum rotation showed on abdominal computed tomography (CT).

The patient was an 84-year-old man who was admitted to the emergency room with a 24-h history of acute colicky abdominal pain. He denied other signs or symptoms. The relevant surgical history included old appendectomy and inguinal hernioplasty. The patient's medical history includes coronary artery disease and atherosclerosis, despite an otherwise healthy status. The patient was hemodynamically stable and in good general condition. Abdominal palpation led to pain in the left lower abdominal quadrant with no signs of peritoneal irritation. Abdominal computed tomography (CT) revealed small-bowel loops with multiple air-fluid levels and dilated stomach with a large air-fluid level (Fig. 1; Fig. 2). Laboratory tests showed no significant alterations.

**Fig. 3:**
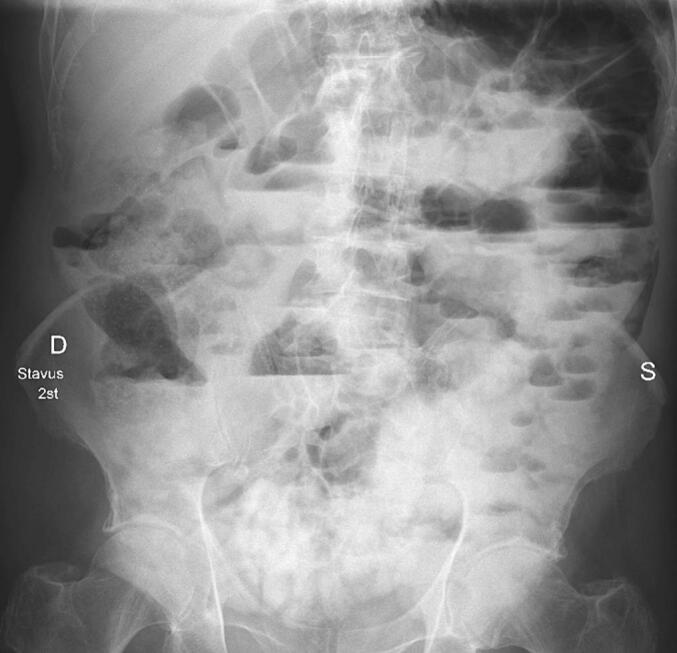
Examination with an oral water-soluble contrast after 2 h.

**Fig. 4:**
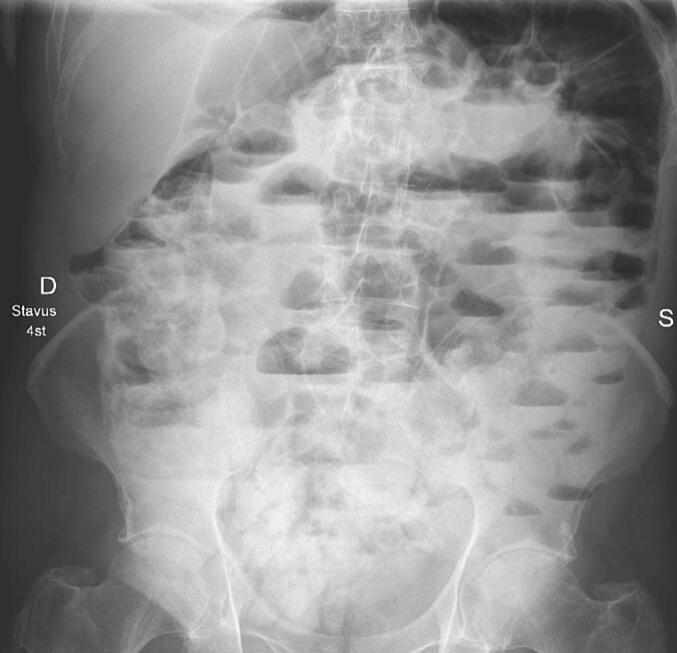
Examination with an oral water-soluble contrast after 4 h.

Decompression with a nasogastric tube was performed, and 1250 mL of light yellow fluid was evacuated. However, the symptoms persisted and he was admitted to the surgery department for further diagnostic testing, observation, and treatment. Despite stable vital signs, the patient experienced mild residual pain during palpation without abdominal distension. The patient stated that he periodically experienced mild abdominal pain for most of his adult life. Examination with an oral water-soluble contrast revealed small-bowel obstruction which remained unresolved over several hours of observation, leading to the suspicion of adhesive small-bowel obstruction (Fig. 3; Fig. 4). After receiving information regarding diagnostic laparoscopy and the potential for further surgical treatment based on intraoperative evaluation, the patient provided consent to the procedure.

**Fig. 5:**
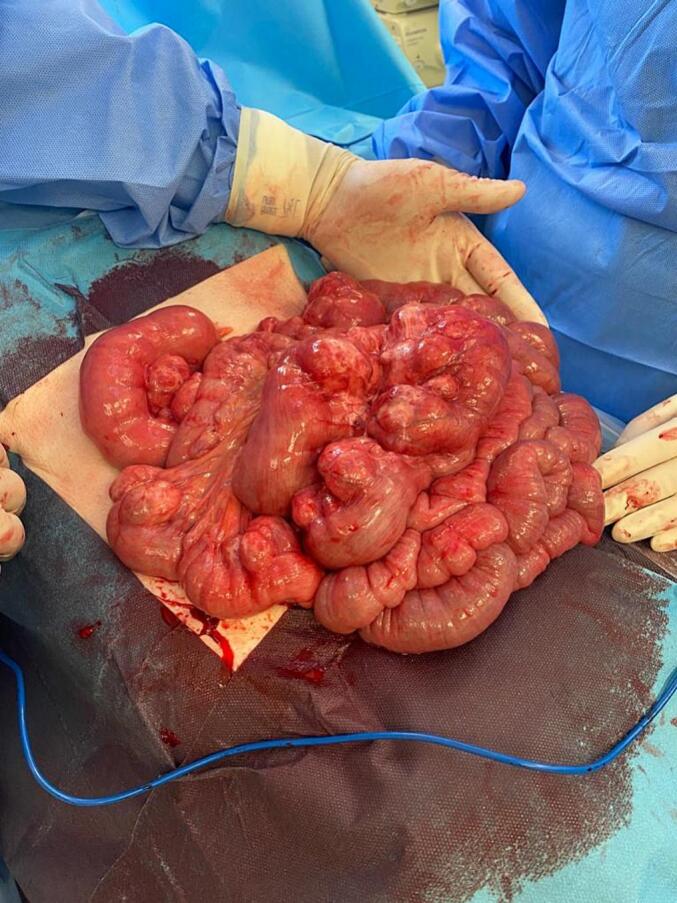
Extensive diverticulosis in the proximal 2-m section of the jejunum, starting approximately 20 cm from Treitz's ligament.

Laparoscopy revealed multiple diverticula in the proximal small intestine with distinctly dilated jejunal loops. Given the extent of the disease and the high iatrogenic injury risk, the procedure was converted to open surgery. A midline laparotomy was performed, and chronic rotation of the mesentery (volvulus) was observed. Derotation of the small intestinal loops was performed. Further examination revealed extensive diverticulosis in the proximal 2-m section of the jejunum starting approximately 20 cm from Treitz's ligament (Fig. 5). Chronic fibrous adhesions and strictures were also observed in the mesentery and the small intestine. The affected small bowel was resected to prevent recurrent volvulus and other complications, such as diverticulitis, perforation, and bleeding. Following the resection of the jejunal segment with multiple large diverticula, side-to-side entero–entero anastomosis was performed. No diverticula were observed in the remaining bowel.

The postoperative period was uneventful, and the patient was discharged from the hospital on postoperative day 6. The histopathologic evaluation of the resected material confirmed substantial jejunal diverticulosis with chronic fibrosis, adhesions, and strictures.

## 3 Discussion

Diverticular disease is markedly less common in the small bowel than in the colon [3] its prevalence ranges from 0.06 % to 4.60 % and increases with age [4]. Approximately 75 % of small-bowel diverticula are located in the proximal jejunum, followed by the distal jejunum (20 %) and ileum (5 %) [5]. Jejunal diverticulosis may be found incidentally but is often identified due to complications [6]. The present case was complicated with mesentery volvulus, which led to small-bowel obstruction, a nuanced issue in emergency abdominal surgery. Caregivers should follow the *nil per os* practice for these patients, and vital signs and urinary output should be monitored for acute changes.

The timing of surgical intervention varies among abdominal surgeons. Surgery is typically chosen for patients with suspected complications, such as peritonitis, who fail to improve with conservative management or in whom the contrast agent fails to reach the colon during contrast studies [7]. Other complications include acute diverticulitis, obstruction, intussusception, gastrointestinal bleeding, anemia, perforation, fistula formation, intra-abdominal abscess, adhesion, and peritonitis [8,12]. The present patient might have remained undiagnosed in the absence of complications. Many patients with jejunal diverticulosis have a history of diffuse abdominal pain, excessive flatulence, bloating, or reduced appetite, vomiting, malnutrition, weakness, and weight loss [6,12]. Iron deficiency anemia and megaloblastic anemia are often linked to malabsorption and vitamin deficiencies, which can result from issues like unsynchronized peristaltic movement, diverticulum enlargement, and bacterial overgrowth. Gastrointestinal bleeding from the diverticulum may explain the patient's non-severe anemia. Nevertheless, bleeding from gastrointestinal tract may manifest with greater severity in other patients [9]. However, these symptoms remain nonspecific and mimic many abdominal pathologies.

The preoperative diagnosis of jejunal diverticulosis remains challenging for both clinicians and radiologists [1]. Jejunal diverticulosis is an infrequent diagnosis in patients undergoing abdominal CT. In one study, jejunal diverticulosis was described in the original CT reports in only 2 of 28 patients (7 %) undergoing barium examination. The challenge in detecting jejunal diverticulosis on CT images is likely related to a combination of factors, including the subtle criteria used to differentiate diverticula from gas- or fluid-filled small-bowel loops and the fact that jejunal diverticulosis is an uncommon cause of abdominal symptoms. Therefore, radiologists may not consider jejunal diverticula on CT images even in cases where the diverticula are causing symptoms [10]. In the present case, abdominal CT and examination with oral water-soluble contrast revealed small-bowel obstruction without any signs of jejunal diverticulosis and extensive jejunal diverticulosis was observed only during laparotomy after diagnostic laparoscopy.

Laparotomy remains the gold standard for the diagnosis of asymptomatic and complicated diverticula [1]. In the absence of symptoms, there is insufficient evidence to justify treatment for diverticula. In instances of diverticulitis, antibiotic therapy and bowel rest is indicated. In the event of significant complications, such as perforation, obstruction and massive bleeding, surgical intervention becomes necessary [8]. Histopathologic evaluation is necessary because tumors can be misdiagnosed as diverticula. For instance, aggressive fibromatosis has been misdiagnosed as jejunal diverticulum with inflammation [11] and gastrointestinal stromal tumor was reported in a jejunal diverticulum [12]. This case report should serve as a reminder for surgeons to be cognizant of the signs of uncommon conditions, such as jejunal diverticulosis, which may be observed during imaging, diagnostic endoscopy, or histopathologic analysis.

In conclusion, complicated jejunal diverticulosis can be a challenging diagnosis compared to colonic diverticulosis. The incidence of jejunal diverticulosis with nonspecific symptoms is low, as evidenced by the very few number of reported cases. Jejunal diverticulosis should be included in the differential diagnosis of acute abdomen, given that early recognition and intervention can prevent mortality.

## Ethics approval

Ethical approval was obtained from The Riga Stradins University Research Ethics Committee (approval no: 2-PĒK-4/599/2023).

## Funding

This study did not receive any specific grant from funding agencies in the public, commercial, or not-for-profit sectors.

## CRediT authorship contribution statement

All the authors participated in the manuscript and validated the final version of the manuscript.

## Guarantor

Sarlote Agate Vanka.

## Declaration of competing interest

All authors declare that there is no conflict of interest.

## Data Availability

All data generated or analyzed during this study are included in this published article.
